# The Neuro-endocrinological Role of Microbial Glutamate and GABA Signaling

**DOI:** 10.3389/fmicb.2016.01934

**Published:** 2016-11-30

**Authors:** Roberto Mazzoli, Enrica Pessione

**Affiliations:** Laboratory of Biochemistry, Proteomics and Metabolic Engineering of Prokaryotes, Department of Life Sciences and Systems Biology, University of TorinoTorino, Italy

**Keywords:** inter-kingdom communication, gut–brain axis, gut microbiota, enteric nervous system, enteroendocrine cells, neuroactive molecules, mood, anxiety

## Abstract

Gut microbiota provides the host with multiple functions (e.g., by contributing to food digestion, vitamin supplementation, and defense against pathogenic strains) and interacts with the host organism through both direct contact (e.g., through surface antigens) and soluble molecules, which are produced by the microbial metabolism. The existence of the so-called gut–brain axis of bi-directional communication between the gastrointestinal tract and the central nervous system (CNS) also supports a communication pathway between the gut microbiota and neural circuits of the host, including the CNS. An increasing body of evidence has shown that gut microbiota is able to modulate gut and brain functions, including the mood, cognitive functions, and behavior of humans. Nonetheless, given the extreme complexity of this communication network, its comprehension is still at its early stage. The present contribution will attempt to provide a state-of-the art description of the mechanisms by which gut microbiota can affect the gut–brain axis and the multiple cellular and molecular communication circuits (i.e., neural, immune, and humoral). In this context, special attention will be paid to the microbial strains that produce bioactive compounds and display ascertained or potential probiotic activity. Several neuroactive molecules (e.g., catecholamines, histamine, serotonin, and trace amines) will be considered, with special focus on Glu and GABA circuits, receptors, and signaling. From the basic science viewpoint, “microbial endocrinology” deals with those theories in which neurochemicals, produced by both multicellular organisms and prokaryotes (e.g., serotonin, GABA, glutamate), are considered as a common shared language that enables interkingdom communication. With regards to its application, research in this area opens the way toward the possibility of the future use of neuroactive molecule-producing probiotics as therapeutic agents for the treatment of neurogastroenteric and/or psychiatric disorders.

## Communication Signals through Different Biological Kingdoms

Living organisms exchange information through systems that are based upon signal-receptor interactions. The specific sense organs of higher animals have evolved to perceive signals, but the whole living world, including protozoans, plants, fungi and bacteria, efficiently communicates by exchanging information at a molecular level. Some universally sensed signals and widespread receptors can be found in different kingdoms.

It has been suggested that ghrelin-like molecules, i.e., those structurally related to the appetite-stimulating lipopeptide hormone which was first isolated from the gastrointestinal tract of rats (Kojima et al., 1999), are ubiquitously present in living organisms (Aydin et al., 2006). These molecules have been found in microorganisms (i.e., viruses, archaea, and phototrophic bacteria), animals and plants, where they fulfill different functions (e.g., inhibition of apoptosis), especially those connected with food intake and cell proliferation (Aydin, 2007). For instance, a structural homolog (*N*-octanoyl homoserine lactone) of ghrelin is produced by Gram-negative bacteria as a quorum sensing (QS) autoinducer and cell-to-cell communication molecule, which is also involved in food-searching (Aydin et al., 2006).

Bacteria produce a huge range of compounds that are involved in inter-microbial and host–microbe relationships. It is well-known that QS peptides, once interpreted as specific bacteria–bacteria communication signals (and regulating phenotypes such as competence, sporulation, bioluminescence, biofilm formation, bacteriocin/toxin production) also have metal (e.g., iron) binding and antibiotic properties, that is, additional functions which can promote the improvement of human health (Schertzer et al., 2009). It has been established that antimicrobial pigments, such as *Serratia* spp. prodigionines (active against fungal and protozoan infections), can also control cancer and immunity (Williamson et al., 2006). So, a single signal can be detected by different cellular systems. “Dark side” examples also exist, as in the case of farnesol, a QS-like molecule synthesized by the yeast *Candida albicans*. Farnesol controls yeast/mycelium transition, but also triggers the disruption of human erythrocytes, and interferes with cytokine expression (Hornby et al., 2001; Navarathna et al., 2007). The extremely long coevolution of host and colonizing microbes can account for these inter-kingdom effects.

The neurochemicals and their related receptors that are found in mammals are far from being an exclusive to these organisms, and are widely dispersed throughout nature, even in microorganisms and plants that do not have nervous systems. For instance, the stress-related neuro-endocrine hormone family of catecholamines has also been demonstrated in fish, insects, plants and bacteria (Lyte, 2011). Microorganisms are able to produce a wide variety of neurochemicals. This feature has been studied extensively by scientists engaged in food safety, since the presence of neuroactive molecules in food has been found to be responsible for cases of poisoning, such as the “cheese reaction” and the “scombroid syndrome” (related to food containing large amounts of tyramine and histamine, respectively) (Pessione et al., 2005, 2009). It has been suggested that at least some molecular neurotransmitters and/or neuromodulators and/or neurohormones have been conserved or shared during co-evolution as the “words” of a common language, thus allowing communication between phylogenetically very distant organisms (Lyte, 2011). It has been hypothesized that such a common language contributes to the homeostatic regulation of gut microbiota and possibly to the functioning of the brain and behavior (Lyte, 2011).

## Microbial Neuro-Immuno-Endocrinology: How Gut Microbiota and the Host Cross-Talk

Microbial neuro-endocrinology is a branch of microbiology which has recently arisen, and it refers to the bidirectional communication that exists between commensal or parasite microbiota and the host. Although, some reports refer to the ability of bacteria to control the level of neurotransmitters through Toll-like receptors (TLRs) and heat-shock proteins (Sivamaruthi et al., 2015), this form of communication may occur directly by means of neurochemicals.

The first evidence that bacteria could respond to mammalian neuro-endocrine hormones dates back to 1929. These studies reported the effect of neuro-hormones, and in particular those belonging to the catecholamine family, on enhancing the pathogenic phenotype of several bacterial strains, such as *Clostridium perfringens*, during *in vivo* infections (Lyte, 2011). At that time, these observations were interpreted to mean that neurochemicals suppressed local immunity and thus favored the rapid unimpeded growth of infectious microbes (Lyte, 2011). However, evidence that prokaryotes can directly sense and respond to neurochemicals (e.g., altering their growth and/or virulence potential) has started to accumulate since the early 1990s. As an example, it is known that some intestinal molecules, such as serotonin [5-hydroxy tryptamin (5-HT)], can modulate the pathogenic potential of *Pseudomonas fluorescens* by affecting its motility and pyoverdin production, but without affecting its growth (Biaggini et al., 2015). Currently, this feature is not considered rare, but is widespread over a high number of diverse microorganisms (Lyte, 2011).

These findings have gained a great deal of attention in light of the parallel emerging evidence of the so-called microbiota-gut-brain axis, which allows bidirectional communication between the gut microbiota and the central nervous system (CNS) of animals, including humans (Cryan and Dinan, 2012). It has been reported that gut microbiota can control the tryptophan metabolism of the host by enhancing the fraction of tryptophan available for the kynurenine route and decreasing the amount available for 5-HT synthesis (O’Mahony et al., 2015). These authors suggested that the deficiencies observed in the serotonergic system of the elderly could be due to quantitative and qualitative modifications of gut microbiota during aging (O’Mahony et al., 2015). The cognitive and behavioral functions of germ-free mice can be altered following colonization with microbiota from a different mouse strain, and this can lead to a similar increased exploratory behavior to that of the donor mouse strain (Bercik et al., 2011b).

The observation of psychiatric co-morbidities in various chronic inflammatory intestinal disorders has been interpreted by some authors as further evidence of the influence of gut microbiota on the CNS (Bercik et al., 2012; Cryan and Dinan, 2012). Altered gut microbiota has been observed in individuals affected by severe psychiatric disorders, such as autism (Adams et al., 2011; Williams et al., 2011). Studies on animals infected by pathogenic bacteria, or treated with probiotic or antibiotic agents have suggested that gut microbiota may play a role in the genesis of multiple sclerosis, anxiety and depression (Berer et al., 2011; Cryan and Dinan, 2012). However, many psychiatric co-morbidities are thought to be the result of perinatal infections, and should be interpreted within the context of altered predisposition rather than microbiota-CNS communication (Buka et al., 2008; Eßlinger et al., 2016; Gilman et al., 2016). Since clear evidence of the role of gut microbiota in the development of these syndromes is still lacking, this subject is still the topic of debate.

However, all these reports have contributed to the concept of the microbiota-gut-brain axis emerging as a “circular” interactive network consisting of multiple (immune, neural, and endocrine) interaction mechanisms. On one hand, the role of the psychological status in the pathogenesis of/recovery from infectious disease is recognized within the medical community (Lyte, 2011). On the other hand, these studies have revolutionized the traditional conception of human–microbiota relationships, and hopefully opened fascinating perspectives as regards the balancing of microbiota through the application of probiotics as adjuvant agents in both neuro-enteric and psychiatric disorders (Mazzoli, 2014). However, it should be pointed out that, due to their extreme complexity and several confounding factors, the understanding of the molecular mechanisms by which intestinal microbiota can influence gut–brain communication or respond to human-derived signals is still at its very beginning. And it is also for this reason that the efficacy of probiotics as therapeutic agents in psychiatric disorders is still a subject of massive debate within the circle of experts (Bron et al., 2011; Kelly et al., 2015; Santocchi et al., 2016).

### The Gut–Brain Axis

The gut–brain axis defines a bidirectional communication system that connects the GI tract with the CNS through multiple pathways involving neural, endocrine, and immune cells. The gut brain axis allows the CNS to regulate GI functions, including motility and secretion, and the GI tract to signal sensations such as hunger, pain, or discomfort to the CNS (Julio-Pieper et al., 2013). However, new complex emotional (affective mood), cognitive (memory formation) and behavioral functions (food intake) are beginning to be discovered (Berntson et al., 2003), and bacteria could play a role in this scenario (Cani and Knauf, 2016). Direct evidence that gut stimuli can affect emotional states includes the fact that the intragastric infusion of fatty acids in humans has been shown to reduce the brain response to experimentally induced sad emotions (Van Oudenhove et al., 2011).

The reciprocal influence between the GI tract and the CNS is sustained by the enteric nervous system (ENS) (Furness, 2006; Cryan and Dinan, 2012), which is considered as the third branch of the autonomic nervous system and consists of about 200–600 million neurons. Owing to its high number of neurons, complexity, and similarity in signaling molecules with the brain, ENS has also been referred to as the “second brain” (Gershon, 1998). ENS interfaces with the gut-associated lymphoid tissue (GALT, which contains more than two-thirds of the body’s immune cells) and with 1000s of entero-endocrine cells (EECs, which contain more than 20 identified hormones). Furthermore, the GI tract is directly innervated by the CNS through spinal and vagal afferents (Mayer, 2011). For this reason, gut-to-brain communications can occur through three main routes: (i) direct perception of stimuli by primary afferent neurons (belonging to the ENS or the CNS); (ii) immune system mediated connections; (iii) EEC mediated connections.

#### Direct Neuronal Perception of Gut Chemical Stimuli

It is generally believed that the afferent terminals that innervate the gut cannot sense luminal chemosignals directly, but can through intermediate cells (e.g., EECs, immune cells) (Mayer, 2011). Under physiological conditions, protein-sized molecules cannot pass the intestinal epithelium through the paracellular pathway. However, lipophilic and small hydrophilic compounds of up to 600 Da can cross the intestinal barrier through multiple routes, e.g., through transcellular (namely, passive diffusion into the lipid bilayer and/or small aqueous pores) and paracellular routes (Keita and Söderholm, 2010). It can therefore be hypothesized that small neuroactive compounds may also use the latter pathways to diffuse in the lamina propria, that is, in contact with intrinsic and/or extrinsic neural afferents, or into the portal circulation, and therefore possibly exert extra-intestinal effects. Some experimental evidence can in fact support this hypothesis (Psichas et al., 2015). For instance, FFAR3 receptors for short chain fatty acids (SCFAs) have been detected in submucosal and myenteric ganglia (Nøhr et al., 2013), and the responsiveness of enteric neurons to glucose, amino acids and fatty acids has been demonstrated (Liu et al., 1999; Furness et al., 2013; Neunlist and Schemann, 2014). Furthermore, pattern recognition receptors that are able to bind a variety of microbial antigens have been identified in neural cells, including enteric neurons, which could possibly enable a rapid activation without the intermediation of immune cells (Lim et al., 2016) (for more details, see Bacteria-Immune-Endocrine-Vagal Connections).

#### Neuroimmune Connections

About 70% of the body’s immune cells are present within the GALT (Mayer, 2011). The existence of close connections between afferent nerve terminals and immune cells (e.g., plasma cells, eosinophils, and mast cells) within the gut mucosa has been well-established (Keita and Söderholm, 2010; Mayer, 2011). For example, the terminals of some vagal afferents can respond to a variety of neuroactive compounds secreted by lymphocytes and mast cells, such as histamine, 5-HT, prostaglandins and various cytokines (Keita and Söderholm, 2010). Furthermore, neuropeptide receptors have been identified in mast cells, thus suggesting bi-directional communication between the nervous and immune systems (Keita and Söderholm, 2010).

#### Neuro-endocrine Connections

Entero-endocrine cells account for less than 1% of epithelial cells in the gut; nonetheless, they constitute the largest endocrine organ of the body (Mayer, 2011). More than 20 different types of EECs have been identified, and these differ according to the type(s) of regulatory peptides (e.g., glucagon-like peptides, GLPs, pancreatic peptide YY, PYY, cholecystokinin, CCK, secretin) or bioactive molecules that they secrete. EECs regulate digestive functions through ENS circuits, and communicate with the CNS (e.g., with the hypothalamus), either directly, i.e., through endocrine pathways, or through paracrine signaling to vagal afferents (Raybould, 2010). Although, EECs and afferent neurons have long been thought to communicate indirectly, i.e., through neuropeptides released by EECs, a recent study has also demonstrated direct synaptic contacts between EECs and neurons (Bohórquez et al., 2015). Experimental evidence has reported that suggests EECs could act as both pre-synaptic (to communicate gut feelings to the nervous system) and post-synaptic (e.g., for the possible modulation of the responsiveness of EECs by efferent neurons) elements.

#### Immune-Endocrine Connections

Immune and enteroendocrine pathways are not distinct routes of the gut–brain axis, but can influence each other to some extent. Indeed, the release of interleukin-4 and 13 from CD4+ T cells, in a mouse gut inflammation model has been reported to increase cholecystokinin secretion in EECs (McDermott et al., 2006). Enterochromaffin cells (ECs) have been described as being able to modulate gut inflammation through 5-HT signaling (Mawe et al., 2009).

### Bacteria-Immune-Endocrine-Vagal Connections

Studies in germ-free animals have underlined the importance of gut microbiota in the early-life development of the gut–brain axis, with particular reference to the hypothalamic-pituitary-adrenal axis (Sudo et al., 2004). Pattern recognition receptors such as TLRs, which are transmembrane proteins that are able to recognize bacterial envelope components, such as lipopolysaccharide (LPS) and lipoteichoic acids (Medzhitov, 2007), have been detected in a number of neuronal cells which could potentially mediate the direct neuronal sensing of microbial antigens (Lim et al., 2016). TLR4, which can be activated by bacterial LPS, has been detected in the nodose ganglion of the vagus nerve of rats (Hosoi et al., 2005). TLRs 3, 7, and 4 are expressed in the myenteric and submucosal plexus of the murine intestine, the human ileum and the lower dorsal root ganglia (Barajon et al., 2009). Similar results were also obtained by Qi et al. (2011), who detected the expression of TLRs 3, 7, and 9 in both human and murine dorsal root ganglion neurons. High mRNA levels of TLRs 1, 4, 5, and 6 have been found in the colonic dorsal root ganglion neurons of mice (Ochoa-Cortes et al., 2010). Furthermore, a number of studies have reported the direct excitation of neurons by LPS (Lim et al., 2016). However, in yet other studies the stimulation of pattern recognition receptors of neurons did not cause any direct excitation, but instead sensitized them and potentiated their excitability (Lim et al., 2016). Nonetheless, these researches have highlighted the existence of other possible pathways for bacteria sensing and communication pathways between the nervous and the immune systems.

Microbial exocellular polysaccharides (EPSs) are essential for protecting bacteria from the host immune response, but also for interacting with intestinal mucosal cells, including epithelial cells and EECs, thus inducing the release of molecular messengers that are able to modulate neural signaling or directly act on primary afferent axons (Forsythe and Kunze, 2013).

Mucosal immune cells are able to distinguish commensal from pathogenic bacteria (Artis, 2008) through TLRs, which are able to detect microbial antigens. Similarly, dendritic immune cells (extending their dendrites between epithelial cell tight junctions so as to directly sense the luminal environment) and some EECs can respond to the presence and activity of intraluminal microbial organisms through TLRs (Mayer, 2011). The intestinal infusion of *Escherichia coli* proteins has been reported to increase the secretion of anorexigenic GLP-1 and PYY, thus providing direct evidence of the ability of the gut microbiota to control appetite (Tennoune et al., 2014; Breton et al., 2016). In addition, EECs possess G-protein-coupled receptors that are able to sense microbial metabolites, such as amino acids (for example glutamate) (Bezençon et al., 2007), protein hydrolysates (Choi et al., 2007) and both long and short fatty acids (Tanaka et al., 2008; Nøhr et al., 2013). The expression of GPR41 receptor of SCFAs has been detected in several types of EECs (e.g., those secreting CCK, ghrelin, gastrin, GLP-1, PYY, neurotensin, secretin) throughout the GI tract of mice, from the stomach to the colon (Nøhr et al., 2013). Both the GPR41 and 43 receptors of SCFAs are expressed by enteroendocrine L-cells (Nøhr et al., 2013). Their stimulation promotes the secretion of GLP-1 and PYY (Samuel et al., 2008; Tolhurst et al., 2012; Nøhr et al., 2013). Furthermore, gut microbiota indirectly regulates the release of GLP-1, GLP-2 and PYY by modulating the differentiation of stem cells into EECs, and thus modifying the number of GLPs and PYY secreting L-cells (Everard et al., 2011). Another group of gut microbiota metabolites, namely indole and tryptamine derived from tryptophan, are known to modulate gut hormone release. Indole triggers GLP-1 secretion by enteroendocrine L-cells (Chimerel et al., 2014). Tryptamine induces ECs to secrete 5-HT (Takaki et al., 1985). Gut microbiota also contributes to regulating sensitivity to leptin, the so-called “satiety hormone” (Schéle et al., 2013).

Several microbial metabolites that display neuroactive properties have been described: (i) gaseous molecules, such as carbon monoxide, hydrogen sulfide and nitric oxide (Bienenstock and Collins, 2010); (ii) SCFAs such as *n*-butyrate, propionate, and acetate (Engelstoft et al., 2008; Nicholson et al., 2012); (iii) amines, such as putrescine, spermidine, spermine and cadaverine, which have been shown to be involved in CNS responses to stress (Bienenstock and Collins, 2010).

Curiously, bacteria can produce a wide range of molecules which mimic human hormones. For instance, muramyl dipeptide (similar to serotonin) (Masek and Kadlec, 1983) and indole (similar to melatonin) (Norris et al., 2013) can both cause sleep and drowsiness, while LPS from Gram-negative bacteria can directly act on thyroid cells, *via* type 4 TLRs, and up-regulate thyroglobulin gene expression (Nicola et al., 2009).

On the other hand, the direct response of bacteria to some of the regulatory peptides/neuroactive molecules that are secreted by EECs and/or the human nervous system has been demonstrated, which indicates the presence of neuromodulator/neurotrasmitter receptors in the bacterial envelope (Lyte, 2011). For example, the stress hormones epinephrine and norepinephrine increase the *in vitro* growth of *E. coli* by more than four orders of magnitude (Freestone et al., 2002) and the *Clostridium*/*Bacteroides* ratio in the human gut (Bailey et al., 2011). A recent report has demonstrated that *Vibrio cholerae* can respond to epinephrine and norepinephrine (enhancing the growth rate, swimming motility, and production of virulence factors such as iron sequestrating phenotype) by means of specific sensor proteins (Halang et al., 2015).

**FIGURE 1:**
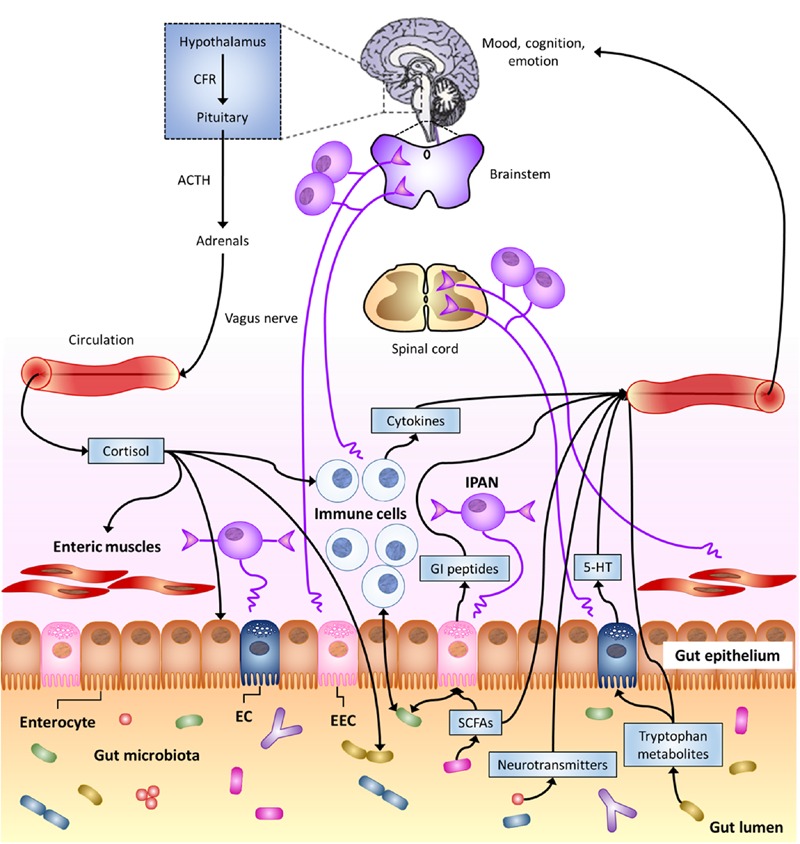
**The Microbiota-Gut–Brain Axis.** Bi-directional communication between gut microbiota and the central nervous system (CNS) can occur through either direct or indirect multiple pathways. These include endocrine, immune, and neural mechanisms (see text). Here, special focus is given to gut-to-brain communication. 5-HT, serotonin; ACTH, adrenocorticotropic hormone; CRF, corticotropin-releasing factor; EC enterochromaffin cell; EEC, enteroendocrine cell; GI, gastro-intestinal; IPAN; intrinsic primary afferent neuron; SCFAs, short-chain fatty acids (modified from Cryan and Dinan, 2012).

Actually, the most forefront hypotheses consider that brain and gut commensal bacteria communicate with each other through shared chemical mediators, and this is part of the homeostasis mechanisms that help to maintain gut microbiota stability and possibly brain functions and behavior (Bienenstock and Collins, 2010; Lyte, 2011). This neurochemical-mediated “two-way street” (**Figure 1**) is one of the principles that supports the microbial endocrinology construct (Lyte, 2011).

## Bacterial Production of Neuroactive Molecules

The molecules that have assessed neuroactive properties produced by bacteria are reported in **Table 1**. This list is far from being exhaustive, and will likely include a progressively higher number of strains and molecules as the results of a greater number of studies become available. The bacteria that are able to produce catecholamines include *Bacillus* spp. (producing dopamine), *Escherichia* spp., and *Bacillus* spp. (producing noradrenalin) (Lyte, 2011). The presence of the complete biosynthetic pathway for catecholamines in these organisms has raised the hypothesis that cell-to-cell signaling in vertebrates may be the result of a late horizontal gene transfer from bacteria (Lyte, 2011).

**Table 1:** Main neuroactive amines and amino acids released by bacteria.

Genus/species	Bioactive molecule and (precursor)	Physiological effects	Reference
*Lactobacillus* spp., *Enterococcus*	Histamine (His)	Hypotension, allergies	Pessione et al., 2005
*Enterococcus faecalis*	Tyramine (Tyr)	Hypertension, headaches	Pessione et al., 2009
*Enterococcus faecalis*	β-phenylethylamine (Phe)	Appetite/satiety control, mood control	Shimazu and Miklya, 2004
*Bacillus*	Dopamine (Tyr)	Multiple	Lyte, 2011
*Bacillus, Escherichia coli*	Nor-adrenaline (Tyr)	Multiple	Lyte, 2011
*Bifidobacteria*, LAB	Melatonin (Trp)	Smooth muscle relaxation, Sleep/wake cycle regulator	Wong et al., 2015
*Lactobacillus bulgaricus, Streptococcus, Escherichia coli*	Serotonin/triptamin (Trp)	Vagal circuit regulation, peristalsis	Matur and Eraslan, 2012
*Corynebacterium glutamycum, Lactobacillus plantarum, Lactobacillus paracasei, Lactococcus lactis*	Glu	Multiple	Tanous et al., 2005; Sano, 2009; Zareian et al., 2012
LAB, *Escherichia coli, Pseudomonas*	GABA (Glu)	Anxiolytic, miorelaxant	Richard and Foster, 2003; Chou et al., 2008; Mazzoli et al., 2010

Strains belonging to *Streptococcus* spp., *Escherichia* spp. and lactic acid bacteria (LAB) are known to synthesize 5-HT (Lyte, 2011; Matur and Eraslan, 2012). It should be underlined that, unlike what is generally believed, the gut (and not the brain) is the main producer of 5-HT in mammals. The ECs of the gut mucosa are the predominant site for the synthesis and storage of 5-HT in humans (Gershon and Tack, 2007). The release of 5-HT occurs in response to chemical stimuli, e.g., the presence of nutrients (e.g., glutamate, glucose), or food-related/bacterial toxins in the intestinal lumen (Mayer, 2011; Kitamura et al., 2012). However, 5-HT produced by gut microbiota could possibly contribute to the overall 5-HT pool in the gut. It is known that 5-HT is involved in the regulation of gut peristalsis, in vagal circuits associated with nausea and vomiting and in the perception of visceral discomfort and pain through spinal afferents (Costedio et al., 2007). For these reasons, 5-HT has been the target of several treatments of gastrointestinal and gut–brain associated disorders such as inflammatory bowel disease, irritable bowel syndrome, post-infectious irritable bowel syndrome, and idiopathic constipation (Costedio et al., 2007).

Several LAB strains are able to synthesize β-phenylethylamine and/or tyramine and/or tryptamine that belong to a group of structurally related neuroactive compounds which are commonly known as trace amines (Mazzoli, 2014). Trace amines (which also include octopamine) are physiologically present in the human nervous system, although in low amounts compared to 5-HT and catecholamines, with which they display close metabolic and neurophysiological relationships (Burchett and Hicks, 2006). Tryptamine production has been detected in *Lactobacillus bulgaricus* (Pessione et al., 2009), while *Leuconostoc* and *Enterococcus* species have been reported to produce both tyramine and β-phenylethylamine (Pessione et al., 2009). It is worth noting that tryptophan decarboxylases have been found in at least 10% of the samples tested from the NIH Human Microbiome Project (Williams et al., 2014). Despite their low amounts, trace amines, such as β-phenylethylamine, could play a significant role as the co-transmitters and neuromodulators (Burchett and Hicks, 2006) that are involved in mood control, appetite/satiety circuits, and attention deficit/hyperactive disorders (Shimazu and Miklya, 2004). Alterations in the trace amine network also seem to be involved in psychiatric syndromes such as bipolar disorder (BD), parkinsonism, and hepatic encephalopathy (Burchett and Hicks, 2006).

Lactic acid bacteria and several other bacteria, such as those that can contaminate fish or shellfish products, produce large amounts of histamine, which is widely known as a mediator of allergy and anaphylaxis but also plays a role as a neurotransmitter in both the CNS and the ENS (Lyte, 2011).

Molecules that control QS communication between bacteria (mainly peptides or acyl-homoserine lactones) have also been shown to be involved in neuronal functioning (Hughes and Sperandio, 2008).

### Bacterial Production of Glutamate and γ-Aminobutyric Acid (GABA)

L-Glutamate (Glu) and γ-aminobutyric acid (GABA) are mainly known for their role as the main neuro-transmitters in the mammalian CNS, with excitatory and inhibitory roles, respectively (Hyland and Cryan, 2010; Julio-Pieper et al., 2013). However, these molecules are widespread in nature and have multiple functions, including plant signaling and communication between bacteria (Dagorn et al., 2013).

Several bacterial strains are able to produce Glu. Coryneform bacteria, such as *Corynebacterium glutamicum, Brevibacterium lactofermentum* and *Brevibacterium flavum*, have been used extensively for the industrial fermentative production of Glu (Sano, 2009). LAB strains belonging to *Lactobacillus plantarum, Lactobacillus paracasei*, and *Lactococcus lactis* are also able to synthesize Glu (Tanous et al., 2005). A recent study has revealed that about 15% of the LAB strains isolated from Asian fermented foods are Glu producers (Zareian et al., 2012).

Both prokaryotes and eukarya synthesize GABA through the decarboxylation of Glu by glutamate decarboxylase (GAD). GAD has been found in both Gram-positive and Gram-negative bacteria, where it is associated with systems that are involved in pH homeostasis and the generation of metabolic energy (i.e., proton motive force) (Pessione, 2012; Tsai et al., 2013). Marine microorganisms (Morse et al., 1980), *E. coli* (Richard and Foster, 2003) and *Pseudomonas* (Chou et al., 2008) can synthesize GABA. Among those microorganisms that are generally recognized as safe or health-promoting, several LAB (e.g., strains belonging to *Lactobacillus, Lactococcus*, and *Streptococcus* genera) and *Bifidobacterium* strains have been reported to biosynthesize GABA (Siragusa et al., 2007; Li and Cao, 2010; Mazzoli et al., 2010; Lyte, 2011). Recently, one *Lactobacillus* strain and four strains of *Bifidobacterium* isolated from the human intestine have been reported to being able to produce GABA (Barrett et al., 2012). Furthermore, a very recent analysis on metagenomic data from the Human Microbiome Project suggests that genes encoding GAD could be present in a significant proportion of human gut microbiota (Pokusaeva et al., 2016). Lactobacilli include the strains with the highest GABA production, although this metabolic ability is more likely strain- rather than genus-related (Li and Cao, 2010).

## Glu/GABA: Receptors, Signaling, and Transport

### Bacterial Targets: Glu and GABA Receptors in Prokaryotes

The role of GABA in the communication between bacteria is in line with the identification of GABA-binding proteins in different prokaryotes. These proteins consist not only of transporters, such as GabP in *E. coli* or *Bacillus subtilis*) (Brechtel and King, 1998; Hu and King, 1998) and Bra in *Agrobacterium tumefaciens* (Chevrot et al., 2006), but also of possible specific receptors (Guthrie and Nicholson-Guthrie, 1989). It has been reported that metabotropic Glu and GABA_B_ receptors, as well as bacterial periplasmic amino acid binding proteins, may have evolved from a common ancestor (Cao et al., 2009). In addition, as reported in the previous section, many bacteria (including those colonizing the human gut) can synthesize GABA, thus suggesting that, as in the case of eukaryotes, GABA might be a conserved and ubiquitous communication molecule.

The fact that GABA has an effect on the physiology of *Pseudomonas* can be supported by several observations. A periplasmic protein showing high affinity for GABA and displaying similar biochemical features to a subunit of the mammalian GABA_A_ ionotropic receptor was identified in an environmental strain of *P. fluorescens* (Guthrie and Nicholson-Guthrie, 1989; Guthrie et al., 2000). Specific receptors for sensing GABA have been found in other Pseudomonads including: the PctC of *P. aeruginosa* and the McpG of *P. putida*, which are involved in GABA chemoreception (Reyes-Darias et al., 2015). Another study has demonstrated that GABA increases the cytotoxicity and virulence of *P. aeruginosa* through a sophisticated modulation of the protein expression (Dagorn et al., 2013). A functional proteomic analysis revealed that six proteins were differentially expressed in *P. aeruginosa* when exposed to GABA (Lyte, 2011). Hence, some bacteria are able to respond to GABA, of both prokaryote and mammalian origin.

A structurally similar ion channel to eukaryotic ionotropic GABA_A_ receptors was identified in the plant pathogen *Erwinia chrysanthemi* (Zimmermann and Dutzler, 2011). The prokaryotic protein is activated by different amines, including GABA, and is modulated by benzodiazepines in a similar manner to its eukaryotic homolog (Zimmermann and Dutzler, 2011; Spurny et al., 2012).

Less information is available on prokaryotic Glu receptors. As far as we know, only a single case of a Glu-activated potassium channel in a bacterium (i.e., GluR0 from *Synechocystis* PCC 6803) has been reported to date (Chen et al., 1999). However, 100 prokaryotic channel proteins with putative Glu binding domains have recently been identified through a bioinformatic study (Ger et al., 2010). Among them, 22 proteins have been found to be homologs of vertebrate ionotropic Glu receptors (Ger et al., 2010).

Based on these findings, it is possible to hypothesize that members of the human gut microbiota can also sense and respond to GABA/Glu, although this has yet to be confirmed through dedicated studies. Furthermore, human-derived or dietary Glu can affect the balance of gut microbial populations by stimulating the growth of certain bacterial species at the expense of others. It is known, for instance, that LAB decarboxylate Glu to GABA in order to create a protonic gradient and enhance metabolic energy. Hence, Glu can constitute a privileged energy source that can be used to expand LAB populations.

### Host-Targets: the Glutamatergic/GABAergic System in the CNS

Glu and GABA are the major excitatory and inhibitory neurotransmitters found in the human CNS, respectively (Julio-Pieper et al., 2013). These compounds are the molecular effectors of a homeostatic neuronal circuit that is characterized by extreme complexity and sophistication at both a physiological and a biochemical level. This system also includes glutamine (Gln), which plays a key role as a non-neuroactive intermediate in the recycling of neurotrasmitters in the brain, mainly Glu and GABA (Soeiro-de-Souza et al., 2015).

**FIGURE 2:**
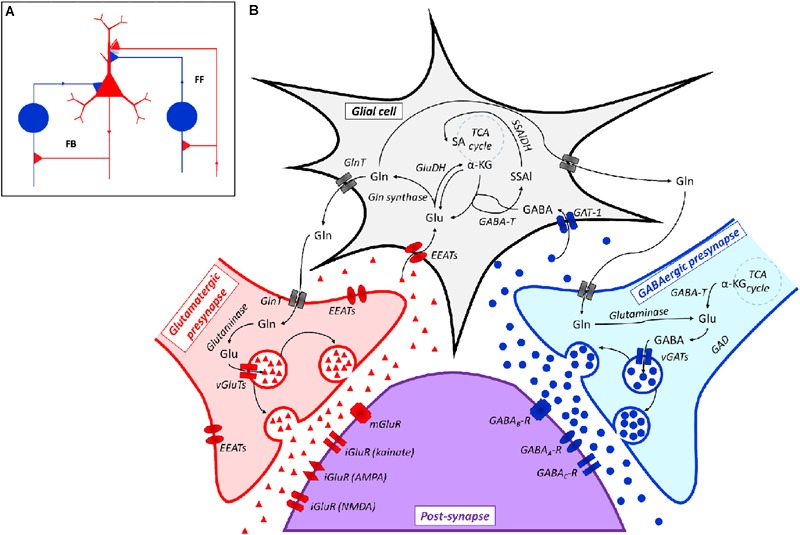
**(A)** Representation of a Glutamatergic/GABAergic neural network, including an excitatory (Glutamatergic) pyramidal neuron (red), inhibitory (GABAergic) neurons (blue) and the related connections. The inhibitory neuron on the left is integrated into a feedback (FB) circuit, while the inhibitory neuron on the right is integrated into a feed-forward (FF) loop. Inhibitory synapses targeted to the soma or dendrites of the pyramidal cell are surrounded by light blue staining, which indicates a background GABA concentration that contributes to the overall inhibition (adapted from Roth and Draguhn, 2012). **(B)** Scheme of the Glutamatergic/GABAergic neurotransmission in the CNS. α-KG, α-keto-glutarate; EEATs, excitatory amino acid transporters; GABA, γ-aminobutyric acid; GABA_A_-R, GABA_A_ receptor; GABA_B_-R, GABA_B_ receptor; GABA_C_-R, GABA_C_ receptor; GABA-T, GABA transaminase; GAD, Glu decarboxylase; GAT-1, GABA transporter 1; GlnT, glutamine transporter; Glu, glutamate; iGluR, ionotropic Glu receptor; mGluR, metabotropic Glu receptor; SSAl, succinic semialdehyde; SSAlDH, succinic semialdehyde dehydrogenase; TCA cycle, the Krebs cycle; vGAT, vesicular GABA transporter; vGluT, vesicular Glu transporter. Symbols: ●, GABA; ▲, Glu.

At a physiological level, this network consists of feed forward (FF) and feedback (FB) neuronal connections in which the main role of inhibitory (i.e., GABAergic) neurons is currently considered as that of balancing the activity of excitatory (i.e., Glutamatergic) neurons (Roth and Draguhn, 2012; **Figure 2A**). Both Glutamatergic and GABAergic neurotransmissions are complex systems in which glial cells (mainly astrocytes), pre- and post-synaptic neurons and a large spectrum of different receptors, transporters and enzymes are involved (Cherlyn et al., 2010; Femenía et al., 2012; **Figure 2B**).

The biochemical relationship between Glu and GABA goes far beyond this reciprocally modulating activity, since Glu and GABA are easily interconverted. Actually, the biochemical reactions involved in GABA-to-Glu-to-Gln conversion are integral features of such a regulatory network, which also involves the central energy metabolism of neurons (Soeiro-de-Souza et al., 2015; **Figure 2B**).

Glu is synthesized *in situ* in the CNS since it cannot pass the blood–brain barrier (BBB) (Hawkins, 2009). One of the main Glu biosynthetic pathways consists of the conversion of glucose (through the Embden-Meyerhof-Parnas pathway and the Krebs cycle) to α-ketoglutarate (α-KG), which is subsequently transaminated (Cherlyn et al., 2010). Glu is accumulated in vesicles and, after suitable stimulation, released to the synaptic space, where it can bind to a panel of Glu receptors (GluRs) that may be found in post-synaptic neurons. GluRs differ on the basis of their mechanism of action (e.g., ionotropic, iGluR, or metabotropic, mGluR, Glu receptors) and pharmacological responsiveness [e.g., *N*-methyl D-aspartate, NMDA, or α-amino-3-hydroxy-5-methylisoxazole-4-proprinoic acid (AMPA) or kainate sensitive receptors]. Ligand-gated ionotropic receptors mediate fast synaptic responses by directly regulating the ion influx. Metabotropic receptors are coupled to G-proteins and modulate slower signal transduction cascades (Hyland and Cryan, 2010; Julio-Pieper et al., 2013). Any excess Glu present in the synaptic cleft is taken up by glial cells or neurons to prevent the prolonged excitation of post-synaptic neurons, which can lead to cell death (Cherlyn et al., 2010). Glu in the glia is either oxidatively deaminated to α-KG [by Glu dehydrogenase (GluDH)], or converted to Gln by Gln synthetase. Gln is released from glial cells, uptaken by pre-synaptic neurons [*via* Gln transporters (GlnT)], and is then converted back to Glu by glutaminase. Apart from its role in neurotransmission, Glu is also involved in other neural processes, such as neuronal development and synaptic plasticity (Cherlyn et al., 2010).

Inhibitory pre-synaptic neurons convert Glu to GABA through GAD and pack it into vesicles *via* vesicular GABA transporters (vGATs). Again in this case, Gln is an alternative Glu source to α-KG. The multiple GABA receptors that are present in post-synaptic neurons include ionotropic (GABA_A_, GABA_C_) and metabotropic (GABA_B_) receptors. Apart from GABA, these receptors are possibly modulated by a number of other compounds, e.g., barbiturates, baclofen, benzodiazepines and steroids (Simeone et al., 2003; Goetz et al., 2007). Any excess unbound GABA is cleared by glial cells through GABA transporters (GAT-1). GABA is then converted by GABA transaminase (GABA-T) into succinic semialdehyde, with concomitant Glu biosynthesis (Sivilotti and Nistri, 1991; Michels and Moss, 2007).

### Glu/GABA: From the Gut to the Brain

As described above, several bacteria, including probiotic bacteria and bacteria that colonize the human GI tract, are able to produce Glu or GABA (Barrett et al., 2012). As far as we know, only one study concerning the modulation of host Glutamate/GABAergic systems by GABA/Glu producing gut-colonizing bacteria is available in the literature (Bravo et al., 2011). A recent study has demonstrated that a GABA-producing *Bifidobacterium dentium* is able to attenuate sensitivity of dorsal root ganglia neurons in a rat model of visceral pain (Pokusaeva et al., 2016). It is worth noting that these few examples refer to the limited number of studies on interaction between gut microbiota and the host nervous system which is currently available. Experimental evidence is likely to grow as a higher number of reports become available. To date, most studies on the Glu/GABA effects on humans concern dietary Glu/GABA.

Glu and GABA can be found in food as natural components or as food supplements. Glu is among the most abundant amino acids (8–10%) found in dietary proteins. Furthermore, monosodium glutamate is employed extensively as a flavor additive in food (Brosnan et al., 2014). In Germany, Glu average intake (comprinsing both free Glu and Glu as protein constituent in foods) of about 10 g/day has been estimated (Beyreuther et al., 2007). In asian consumers this value is likely higher since free Glu intake can reach up to 4 g/day in these countries (Beyreuther et al., 2007). GABA is relatively abundant in plant food, such as brown rice germs and sprouts, spinach, barley and bean sprouts, where GABA concentrations of between 300 and 720 nmol/g dry weight have been detected (Oh et al., 2003), while higher levels are found in fermented foods (Abdou et al., 2006). In recent years, the utilization of GABA as a food supplement has progressively increased in both Asian and Western (USA and Europe) countries (Boonstra et al., 2015). GABA concentration of few micrograms per gram of cecal content has been detected in mice (Pokusaeva et al., 2016).

Hundreds of people have reported the benefits of using GABA-supplemented food, for instance in alleviating anxiety and/or improving sleep quality (Boonstra et al., 2015). Moreover, at least five different studies have reported that the oral administration of GABA or GABA-supplemented food/beverages (corresponding GABA amounts of about 50–100 mg) has had positive effects on human health. These effects include: (i) the reduction of psychological stress in people who performed arithmetic tasks (Nakamura et al., 2009; Kanehira et al., 2011; Yoto et al., 2012); (ii) the reduction of stress in acrophobic subjects exposed to heights (Abdou et al., 2006); (iii) an increased ability to perform prioritized planned actions (Steenbergen et al., 2015).

**FIGURE 3:**
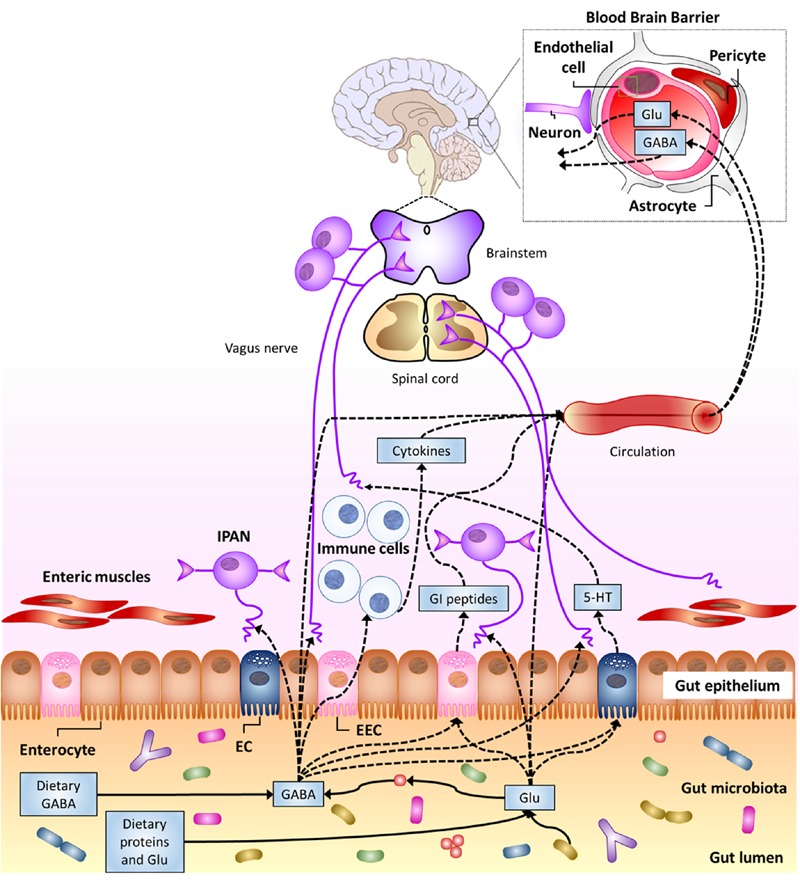
**Potential pathways for luminal Glu/GABA in the gut (of either dietary or gut–microbiota origin) to influence the nervous system (see text). 5-HT, serotonin; EC enterochromaffin cell; EEC, enteroendocrine cell; GI, gastro-intestinal; IPAN; intrinsic primary afferent neuron (from Cryan and Dinan, 2012)**.

Multiple pathways by which gut luminal Glu/GABA may affect the CNS can be hypothesized, including their transport across intestinal and BBB and their sensing through afferent terminals that innervate the GI tract (**Figure 3**). In the next sections, evidence possibly supporting these mechanisms will be illustrated.

#### Direct Influence of the Luminal Glu/GABA in the Gut on the CNS

Multiple transporters that mediate Glu absorption have been found in the apical membrane of GI epithelial cells, mainly in the small intestine, but also in the stomach (Burrin and Stoll, 2009), while little or no transport of amino acids from the lumen to portal blood occurs in the colon (Julio-Pieper et al., 2013). Studies on pigs and rodents have indicated the excitatory amino acid carrier 1 is the most abundant Glu transporter in the mucosa of the small intestine (Burrin and Stoll, 2009). A study performed in infant pigs has indicated that rate of Glu absorption and transport to the portal circulation linearly increases with increasing intraduodenal Glu intake (Janeczko et al., 2007). Portal absorption rate increased from 3.80 μmol kg^-1^ h^-1^for normal Glu intake (i.e., 510 μmol kg^-1^ h^-1^) to 20.46 3.80 μmol kg^-1^ h^-1^ for piglets infused with 3.5-fold increased Glu intake (Janeczko et al., 2007). Glu is one of the main nutrients for enterocytes. Several studies on different animal models, including pre-term infants and human adults, have agreed on that most Glu present in the GI lumen is oxidized to CO_2_ or, secondarily, converted to other amino acids by the gut mucosa (Burrin and Stoll, 2009). Only a small percentage (between 5 and 17%, depending on the studies) of the ingested Glu is transported into the portal circulation but this does not generally affect the Glu concentration in the plasma to any great extent (Hays et al., 2007; Brosnan et al., 2014). Furthermore, an increased plasma Glu concentration (following Glu intake) does not necessarily affect the Glu concentration in brain tissues, since it is generally recognized that Glu cannot pass the BBB (Janeczko et al., 2007). A 20-fold (or more) increase in plasma Glu concentration was found necessary to access the brain tissues in rodents (Brosnan et al., 2014). The reaching of such a high Glu concentration in plasma after dietary intake (or Glu biosynthesis by gut microbiota) seems rather unlikely.

As far as GABA transporters are concerned, they are mostly found in the CNS (Gadea and López-Colomé, 2001). However, H^+^/GABA symport across the apical membrane of human intestinal epithelial (Caco-2) cells has been demonstrated (Thwaites et al., 2000). In fact, the hPAT1 H^+^/amino acid symporter, which is thought to mediate the uptake of luminal GABA, is present throughout the human GI tract, from the stomach to the discending colon, with maximal expression in the small intestine (Chen et al., 2003). Furthermore, GABA carriers have been detected in the basolateral membrane of Caco-2 cells (Nielsen et al., 2012). These findings suggest that luminal GABA should be able to cross the intestinal barrier and possibly reach extra-intestinal targets. In fact, orally administered GABA in rats was reported to increased GABA concentration in their blood, with a peak after 30 min (Abdou et al., 2006). Nonetheless, GABA ability to cross the BBB is still controversial (Boonstra et al., 2015). Most early studies, performed between 1950 and 1980, agreed on the impermeability of BBB to GABA, but more recent investigations have reported that GABA may actually cross this barrier, although in small amounts (Boonstra et al., 2015). These discrepancies have been ascribed to the different method of administration (e.g., oral versus injection), or animal model employed. Some studies have also suggested that BBB permeability to GABA could diminish with increasing age (Boonstra et al., 2015). Interestingly, GABA-transporters have been found in the BBB of mice (Kakee et al., 2001). Direct evidence of GABA facilitated transport across the BBB of mice has been reported, although the eﬄux rate from the brain was 17 times higher than the influx rate (Kakee et al., 2001). Unfortunately, no information about the possible presence of similar transporters in the BBB of humans is currently available.

All together, these findings suggest that a direct action of luminal gut Glu and/or GABA on the CNS cannot be excluded, although further *in vivo* studies are required to confirm this hypothesis. These investigations should also take into account that gut and/or BBB permeability are affected by several factors, including stress, diet and gut microbiota (Braniste et al., 2014; Kelly et al., 2015).

#### Sensing of Luminal Glu/GABA in the Gut

Multiple Glu receptor types (including ionotropic, types 1 and 4 metabotropic receptors and heterodimeric, TAS1R1 + TAS1R3, L-Glu taste receptors) have been detected in GI epithelial cells and/or enteric neurons in the stomach, small intestine and colon (Kondoh et al., 2009; San Gabriel et al., 2009; Kitamura et al., 2012). More in detail, mGlu_4_ receptors have been detected in the mucosa of both the gastric antrum and duodenum (Akiba et al., 2009), while both mGlu_4_ and mGlu_7_ receptors have been identified in the colon epithelium (Chang et al., 2005; Julio-Pieper et al., 2010). TAS1R3, a subunit of umami taste receptors, has been found in enteroendocrine L-cells and X/A-like cells (Jang et al., 2007; Hass et al., 2010). This evidence suggests a possible role in luminal Glu sensing. In fact, small intestine luminal L-Glu seems to play a role in the defense mechanisms of duodenal mucosa in rats, by increasing the intracellular pH and mucus gel thickness (Akiba et al., 2009). A role of mGluRs in the human colon in the control of colon peristalsis and electrolyte transport has been described (Julio-Pieper et al., 2013). High levels of mGlu_7_ and mGlu_8_ have been detected in myenteric neurons, where they are possibly involved in the regulation of gut motility (Julio-Pieper et al., 2013). However, results on the ability of gut luminal Glu to directly excite enteric neurons still appear contradictory (Kirchgessner, 2001; Wang et al., 2014). On the other hand, an indirect activation of vagal afferents (i.e., *via* the production and release of nitric oxide and 5-HT) through the administration of Glu to the gastric lumen has been demonstrated (Uneyama et al., 2006). These data strongly suggest a role of ECs in the mediation of luminal Glu sensing. This luminal Glu sensing mechanism stimulates different areas in the CNS, including the cerebral cortex, basal ganglia, limbic system, and hypothalamus (for a comprehensive review, refer to Kondoh et al., 2009), and induces flavor preference in rats (Kitamura et al., 2012).

GABA_B_ receptors are abundantly expressed in the GI tract (Hyland and Cryan, 2010). GABA and its ionotropic and metabotropic receptors are widely distributed throughout the ENS, in both submucosal and myenteric neurons, from the stomach to the ileum (Auteri et al., 2015). In addition, Nakajima et al. (1996) reported the expression of GABA_B_ receptors in cells morphologically similar to EECs, where they co-localized with cells containing somatostatin (in the stomach) or serotonin (in the duodenum). However, a later study failed to detect any positive EEC for GABA_B_ receptors (Casanova et al., 2009). Schwörer et al. (1989) showed that the release of 5-HT by ECs in the small intestine of guinea-pig is modulated by GABA_A_ and GABA_B_ receptors. Involvement of GABA_B_ receptors in modulation of sensitivity of vagal and spinal afferents has been reported (Hyland and Cryan, 2010). Actually, the GABA_B_ metabotropic receptors in the GI tract are thought to regulate several functions, including gut motility and gut-to-brain signaling (Hyland and Cryan, 2010). Experimental evidence of GABA_B_/μ-type opiod receptor interaction, which mediates the synergistic potentiation of the anti-nociceptive action of GABA/morphine co-administration, has been reported (Hara et al., 1999). A similar GABA_B_ receptor-mediated potentiation effect has also been described for drugs used to reduce visceral pain (Hara et al., 2004).

Since GABA receptors have been found in a wide range of immune cells, such as dendritic cells, mast cells and T-cells (Auteri et al., 2015), and are involved in regulating immunological processes, such as the down-regulation of pro-inflammatory cytokine release (Bjurstöm et al., 2008), their role in neuro-immune dialog in the gut has been proposed (Auteri et al., 2015).

It is worth reminding that GABA is produced through Glu decarboxylation in both eukarya and prokaryotes. Hence, GABA-producing microorganisms that are present in the gut may significantly affect luminal Glu/GABA ratio and, therefore, gut signaling.

## Probiotics for the Treatment of Psychiatric Disorders?

As far as its application to the field of probiotics is concerned, microbial neuro-endocrinology addresses the ability of probiotics to both synthesize and respond to neuroactive compounds, and thus to affect the host neurological processes and sometimes the overall physiology of the host. A microbial endocrinology-based approach may be relevant to help understand how probiotics can influence the health of a host and how host can select commensal microbial populations.

A number of studies have provided evidence on the ability of probiotic strains to modulate the mood and stress responses of humans, and to reduce anxiety and depression (Logan and Katzman, 2005; Messaoudi et al., 2011; Arseneault-Breard et al., 2012). As an example, the administration of Bifidobacteria and LAB as probiotic supplements to humans can increase the levels of morning salivary melatonin, thus reducing the symptoms of irritable bowel syndrome (Wong et al., 2015). Actually, most of the currently recognized probiotic strains belong to Bifidobacteria and LAB, with lactobacilli being among the most abundant members of such health-promoting strains (Vaughan et al., 2011). A direct modulation of myenteric neuron activity by unidentified *Bifidobacterium longum* fermentation product(s) displaying anxiolytic effects has also been reported (Bercik et al., 2011a).

Alterations in the Glu/ Gln/ GABA circuits in the CNS have been reported for generalized anxiety disorders, as well as in other psychiatric conditions, such as the major depressive disorder (MDD), BD (also known as manic depressive disorder) and schizophrenia (SZ) (Cherlyn et al., 2010; Femenía et al., 2012; Soeiro-de-Souza et al., 2015). It is worth noting that these complex neurobehavioral disorders affect a significant portion of the overall population throughout the world (e.g., individuals affected by BD or SZ account for about 2–3% of the global population) (Cherlyn et al., 2010). Different classes of drugs addressed to different steps of Glutamate/GABA-ergic neurotransmission (e.g., reducing Glu release, increasing Glu re-uptake, or increasing GABA levels by enhancing GAD or inhibiting GABA transaminase and succinic semialdehyde dehydrogenase) or serendipitously discovered (e.g., valproic acid) have been used in the treatment of such mood disorders (Terbach and Williams, 2009; Femenía et al., 2012). However, drugs frequently have negative secondary effects. For instance, most antidepressants (such as tricyclic antidepressants), while having a positive effect on the mood of humans, do not alleviate and can even aggravate cognitive symptoms, or impair vigilance (e.g., selective 5-HT reuptake inhibitors), and may cause dependence (e.g., benzodiazepines) (Femenía et al., 2012). Finding alternative or adjuvant therapies is therefore a research priority and, in this respect, the use of neuromodulating bacteria or so-called psychobiotics is a fascinating perspective.

Interestingly, the anxiolytic and anti-depressant-like effects of *Lactobacillus rhamnosus* JB-1 ingestion in mice involve a reduction in the plasma corticosterone levels and alterations of both GABA_A_ and GABA_B_ receptor expression patterns in specific brain areas (Bravo et al., 2011). Furthermore, increases in the Glx (i.e., Glu + Gln) and GABA levels in the CNS have been observed after 2 and 4 weeks of *L. rhamnosus* JB-1 treatment, respectively (Janik et al., 2016). These effects were mediated by gut-to-brain communication through the vagus nerve (Bravo et al., 2011). However, whether *L. rhamnosus* stimulates the vagus nerve with its own GABA or induces an endogenous GABA production still has to be clarified.

Modulation of the glutamatergic system in the central amygdala, cortex and hippocampus of mice by gut microbiota has been described (Sudo et al., 2004; Neufeld et al., 2011).

Although, GABA production has been speculated as the key factor in the ability of a *Lactobacillus helveticus*–*B. longum* mixture to reduce anxiety-like behavior(s) (Lyte, 2011), the observed probiotic effects on the psychological health of a host are generally likely the result of multiple interactions with the host (ENS, EEC, GALT). On the other hand, anorexigenic and anxiolytic soymorphin peptides (produced by LAB proteolytic action toward soy betaconglycinine) act *via* the GABA_B_ receptor and, in this overall action, multiple circuits (including 5-HT and dopamine receptors) are involved (Kaneko et al., 2010). A recent investigation have suggested that a GABA-producing *B. dentium* is able to reduce nociception in a rat model of visceral pain (Pokusaeva et al., 2016).

Because of the wide range of potential neuro-active compounds (orexant, anorexant, opioid, opioid antagonist) produced by bacteria and the complex network that exists between them, a deep characterization of the proteome and metabolome of probiotic strains is one key aspect that would help to understand the different effects exerted by each strain on human health. Since not only Glu/GABA receptors but also other circuits can be potential targets for bacterial-derived molecules, it is tempting to hypothesize the use of probiotics that produce bioactive compounds (e.g., GABA, 5-HT, opioid peptides) as a novel frontline strategy for the adjuvant treatment of gastrointestinal, neuroenteric, neurological and psychiatric conditions (Hyland and Cryan, 2010; Lyte, 2011; Reid, 2011).

Together with this optimistic viewpoint and the enthusiasm that these findings have generated in the scientific domain, it is worth stating that a number of issues suggests caution in application of psychobiotics. The comprehension of the mechanisms by which gut microbiota and the nervous system communicate is still at its beginning. Multiple levels of complexity hinder the detailed understanding of this network and these include (but are not limited to) the fact that: the gut–brain axis comprises multiple (i.e., neural, immune, and endocrine) pathways with several reciprocal interactions; multiple molecular mediators, such as GABA and Glu, are involved, which play roles in a number of different biological processes; gut microbiota and even single commensal microorganisms interact with the host through multiple pathways and chemical mediators (e.g., cell envelope components, production/metabolism of neurotransmitters or neuroactive molecules). Therefore, it may be difficult to specifically address health problems with such microbial agents without the risk of unbalancing other pathways. As for other potential therapeutic agents, rigorous evaluation of the potential beneficial effects together with possible negative side effects should be performed for each probiotic/psychobiotic agent.

## Conclusion

The increasing knowledge about gut bacteria–brain bi-directional communication has provided scientific evidence that can explain the popular statement that somebody has made a decision based his/her on gut feeling, but also the saying “we are what we eat,” as it is supposed that diet can affect the microbiota composition of the gut. It is now evident that gut microbiota is able to interfere, through its cellular components or by secreting bioactive compounds, with the gut–brain axis through immune, neural and humoral pathways. As the knowledge of this domain increases, new unexpected and exciting perspectives for both basic and applied science will likely emerge. At the same time, the observed effects of human-derived neuro-active compounds on gut microbiota would seem to suggest that some neurochemicals share a common language that enables interkingdom communication, as proposed by Lyte and Freestone (2010). The ability of a microbial strain to affect the overall patho-physiology of a host thus appears to be the result of a long and sophisticated synergic evolution between gut bacteria and the host.

## Author Contributions

Authors equally contributed to writing the manuscript. EP mainly contributed to describe bioactive molecules produced by bacteria and involved in interkingdom signaling, with particular attention to microbiota-gut-brain axis. RM focused his contribution mainly on Glutamate- and GABA-mediated communication, including Glu and GABA biosynthesis and receptors in both bacteria and humans, gut-to-brain transport and sensing and Glu-ergic/GABA-ergic neural transmission in the CNS.

## Conflict of Interest Statement

The authors declare that the research was conducted in the absence of any commercial or financial relationships that could be construed as a potential conflict of interest.
